# A prospective study of Kaposi's sarcoma-associated herpesvirus and Epstein–Barr virus in adults with human immunodeficiency virus-1

**DOI:** 10.1038/sj.bjc.6603100

**Published:** 2006-05-16

**Authors:** R Newton, L Carpenter, D Casabonne, V Beral, A Babiker, J Darbyshire, I Weller, R Weiss, A Kwan, D Bourboulia, F Munoz, D Lagos, C Boshoff

**Affiliations:** 1 Epidemiology & Genetics Unit, Department of Health Sciences, Area 3, Seebohm Rowntree Building, Heslington, York YO10 5DD, UK; 2 Cancer Research UK Cancer Epidemiology Unit, University of Oxford, Oxford, UK; 3 Department of Public Health, University of Oxford, Oxford, UK; 4 MRC Clinical Trials Unit, University College London, London, UK; 5 Centre for Sexual Health and HIV Research, University College London, London, UK; 6 Wohl Virion Centre, Division of Infection and Immunity, University College London, London, UK; 7 Cancer Research UK Viral Oncology Group, Wolfson Institute of Medical Sciences, London, UK

**Keywords:** Kaposi's sarcoma, non-Hodgkin's lymphoma, Epstein–Barr virus, Kaposi's sarcoma-associated herpesvirus, human immunodeficiency virus

## Abstract

Antibody titres against Kaposi's sarcoma associated herpesvirus (KSHV or human herpesvirus 8 (HHV-8)) and Epstein–Barr virus (EBV) were examined in people who subsequently developed Kaposi's sarcoma and non-Hodgkin's lymphoma, within randomised controlled trials of antiretroviral therapy in adults infected with the human immunodeficiency virus-1 (HIV). For each case of Kaposi's sarcoma (*n*=189) and each case of non-Hodgkin's lymphoma (*n*=67), which developed after randomisation, one control was randomly selected from other trial participants, after matching for age, sex, ethnicity, mode of HIV transmission, type of treatment received and period of follow-up. Using sera taken an average of two and a half years before the diagnosis of cancer, titres of antibodies against KSHV latent (LANA) and lytic (K8.1) antigens and against EBV (VCA) antigens were investigated in relation to subsequent risks of cancer by calculating odds ratios (OR) using conditional logistic regression. Latent antibodies against KSHV were detectable among 38% (72 out of 189) of Kaposi's sarcoma cases and 12% (23 out of 189) of their controls (OR=4.4, 95% confidence intervals (CI) 2.3–8.3, *P*<0.001). The OR for Kaposi's sarcoma increased with increasing antilatent KSHV antibody titre (*χ*^2^_1_ for trend=32.2, *P*<0.001). Lytic antibodies against KSHV were detectable among 33% (61 out of 187) of Kaposi's sarcoma cases and 19% (36 out of 187) of their controls (OR=2.0, 95% CI 1.2–3.4, *P*=0.003) and the OR for Kaposi's sarcoma increased with increasing antilytic KSHV antibody titre (*χ*^2^_1_ for trend=6.2, *P*=0.02). Virtually, all cases and controls had anti-EBV antibodies detected and the OR for non-Hodgkin's lymphoma associated with a doubling of the anti-EBV antibody titre was estimated to increase by a multiplicative factor of 1.3 (95% CI 0.9–1.7, *P*=0.1). Kaposi's sarcoma was not associated with antibody levels against EBV (*P*=0.4) and non-Hodgkin's lymphoma was not associated with antibodies against KSHV (latent *P*=0.3; lytic *P*=0.5). Adjustment for CD4 count at the time of sample collection made no material difference to any of the results. In conclusion, among human immunodeficiency virus infected people, high levels of antibodies against KSHV latent and lytic antigens are strongly associated with subsequent risk of Kaposi's sarcoma but not non-Hodgkin's lymphoma. Antibody titre to EBV does not appear to be strongly associated with subsequent risk of Kaposi's sarcoma or non-Hodgkin's lymphoma in HIV infected people.

The risk of Kaposi's sarcoma and non-Hodgkin's lymphoma is increased in human immunodeficiency virus-1 (HIV) infection (Beral and Newton, 1998). Both of these tumours are also associated with a herpesvirus infection: Kaposi's sarcoma has been consistently linked with Kaposi's sarcoma associated herpesvirus (KSHV or human herpesvirus-8 (HHV-8)), while non-Hodgkin's lymphoma has been linked with another gamma-herpesvirus, the Epstein–Barr virus (EBV) (Boshoff, 1999; Brooks *et al*, 1999). However, there are few data from prospective studies and the role of antibody titres in predicting the subsequent risk of cancer is unclear. The aim of the research described here was to investigate using data collected prospectively, the evolution of antibody responses to KSHV and to EBV in HIV infected individuals, who subsequently developed Kaposi's sarcoma or non-Hodgkin's lymphoma compared to that in HIV infected controls without cancer.

## MATERIALS AND METHODS

### Study population

UK participants in the Concorde (1994), Delta (1996) and Alpha (1996) randomised controlled trials of antiretroviral therapy for HIV infection were followed for the development of AIDS-defining cancers. Cases were all participants who did not have a tumour at trial entry, but who subsequently developed Kaposi's sarcoma (*n*=189) or non-Hodgkin's lymphoma (*n*=67). The diagnosis of cancer was made at the participating clinical centre according to criteria used in each of the three clinical trials and all data used in these analyses were anonymised. No additional histological confirmation of diagnosis was available for the purposes of this investigation, nor were any details of the specific subtypes of non-Hodgkin's lymphoma available. Further details of the individual trials can be found elsewhere (Concorde Co-ordinating Committee, 1994; Delta Co-ordinating Committee, 1996; Alpha International Co-ordinating Committee, 1996).

For each person with incident cancer, a control was randomly selected from trial participants, who had not developed cancer after the same period of active follow-up (methods of follow-up are described in the individual trial reports), after matching by trial, age group (±10 years), sex, HIV transmission group (homosexual, intravenous drug user, haemophiliac, blood transfusion, other blood contact or heterosexual), treatment group used in each trial (zidovudine (AZT) or placebo [Concorde], low-dose or high-dose didanosine ddI [Alpha], AZT or AZT+ddI or AZT+zalcitabine (ddC) [Delta]), ethnicity (Caucasian, Indian subcontinent, West Indian, African, other or mixed) and length of time from entry into the trial until diagnosis of cancer in cases or the same time interval in controls (±3 months).

At least one sample of stored serum was available for all cases and their matched controls. In general, these were taken at least 6 months before the date of diagnosis of cancer in cases, or at the same period of follow-up as the cases to whom they were matched for controls – referred to as pseudo-diagnosis. Sera were stored centrally at −70°C and shipped on dry ice to University College London for antibody analyses. A proportion of cases and controls had two serum samples available for testing, taken at different time points prior to diagnosis.

### Laboratory investigations

All serum samples were tested for antibodies against KSHV and EBV. Assays were performed by investigators, who were blinded to the identity and personal characteristics of the person from whom each sample came and specifically if they were a case or a control. Details of the testing procedure for antibodies against both latent and lytic KSHV antigens are described elsewhere (Newton *et al*, 2003a, 2003b; Bourboulia *et al*, 2004). Briefly, an indirect immunofluorescence assay to detect IgG antibodies against the KSHV latent nuclear antigen (LANA) encoded by orf73 was used. Serum samples that were positive for antibodies against latent KSHV antigens (at a dilution of 1 : 100 or more) were then tested at doubling dilutions, starting at 1 : 100 in order to provide a measure of anti-KSHV antibody titre. For the detection of anti-lytic KSHV antibodies, an adapted multiantigenic peptide (MAP) based enzyme-linked immunoassay against the late phase of KSHV replication K8.1 protein was used (Lam *et al*, 2002). Concordant results were obtained for 78% of 253 controls in whom both assays were conducted.

For the detection of human IgG antibodies to EBV, all but two control samples were analysed by indirect immunofluorescence (IFA) (Akre *et al*, 1999). Viral capsid antigen (VCA) IgG was determined with acetone-fixed P3HR-1 cells (Hinuma *et al*, 1967). Titres were determined in two-fold dilutions from 1/10^1^ to 1/10^4^. Cutoff for positivity was set at titre ⩾40. The titre of the antibody was taken as the dilution where brightness of fluorescence appeared to diminish.

Ramos cells (both KSHV and EBV negative cell lines) were used to test for crossreactivity with antigens from both viruses. No such reactivity was observed.

CD4 T-lymphocyte counts (measured as described in individual studies) at, or within, 3 months, of the date of collection of the sample used in the study, were available for 86% (163 out of 189) of Kaposi's sarcoma cases and for 75% (50 out of 67) of non-Hodgkin's lymphoma cases and their matched controls. For two case–control pairs, there were insufficient sera available for testing for antibodies against KSHV lytic antigens.

### Statistical analyses

Statistical analyses were conducted using STATA software (version 7.0; 2001). Odds ratios (ORs) for cancer in relation to anti-HHV-8 and anti-EBV antibody titres were estimated with 95% confidence intervals (CI) using conditional logistic regression, with and without adjustment for CD4 count (<200, 200–500, >501) at the time of sample collection. Tests for statistical significance of OR and linear trend were derived from likelihood ratio test statistics. All *P*-values are derived from two-sided tests of statistical significance.

The main analyses included all subjects who had at least one sample of serum available, taken at least 6 months before the date of diagnosis of cancer in cases or pseudo-diagnosis in controls. For those subjects with two serum samples available, the first was used. Some additional analyses were restricted to individuals for whom two serum samples were available prior to diagnosis. In relation to KSHV, latent antibody titres were grouped into negative (at a dilution of less than 1 : 100) and positive. Seropositives were further subdivided into low titre (dilutions of 1 : 100 to 1 : 1600), medium titre (1 : 3200 to 1 : 25 600) and high titre (1 : 51 200 or more). These categories were chosen on the basis of results from previous case–control studies of Kaposi's sarcoma in relation to anti-KSHV antibody titres (Sitas *et al*, 1999; Newton *et al*, 2003a, 2003b). Kaposi's sarcoma associated herpesvirus lytic antibodies were grouped into negative (optical density (OD) <0.84); indeterminate (low OD 0.84–1.06) and positive (OD>1.06). The seropositives were further subdivided according to the optical density (medium OD 1.07–1.99 and high OD>1.99). In analyses involving two levels of lytic anti-KSHV antibodies, those with indeterminate values were grouped together with the negatives.

Since the assays against latent and lytic anti-KSHV antibodies are measuring responses to different aspects of the viral life cycle, no attempt was made to combine the results from each one. All but two subjects had detectable antibodies against EBV and relative risks were, therefore, estimated in relation to a doubling of titre.

## RESULTS

Table 1 shows the distribution of Kaposi's sarcoma and non-Hodgkin's lymphoma cases and their controls, according to the variables on which they were matched and to the CD4 count at the time that the first serum sample was taken.

### Kaposi's sarcoma

The analysis of all study participants who had at least one sample of serum available, taken an average of about two and a half years prior to cancer diagnosis (or pseudo-diagnosis in controls) is shown in Table 2 for antibodies against KSHV latent (189 case–control pairs) and lytic (187 case–control pairs) antigens. Among those who developed Kaposi's sarcoma, the prevalence of latent anti-KSHV antibodies was 38% (72 out of 189) and among their matched controls it was 12% (23 out of 189; odds ratio (OR)=4.4, 95% CI=2.3–8.3, *P*<0.001). The risk of Kaposi's sarcoma increased with increasing latent anti-KSHV antibody titre (*χ*^2^_1_=32.0; *P*<0.001). Adjustment for CD4 count at time of sample collection made little difference to the result (*χ*^2^_1_=26.0; *P*<0.001). Among those who developed Kaposi's sarcoma, the prevalence of lytic anti-KSHV antibodies was 33% (61 out of 187) and among their matched controls it was 19% (36 out of 187; OR=2.0, 95% CI=1.2–3.4, *P*=0.003). The risk of Kaposi's sarcoma increased with increasing lytic anti-KSHV antibody titre (*χ*^2^_1_=6.2; *P*=0.02). Adjustment for CD4 count at time of sample collection made little difference to the result (*χ*^2^_1_=4.3; *P*=0.04).

Table 3 shows the OR for Kaposi's sarcoma according to latent and lytic anti-HHV8 antibody status, stratified by the time between sample collection and diagnosis. For both antibody measures, there were no statistically significant differences in the OR for Kaposi's sarcoma, according to the time between sample collection and diagnosis of cancer.

Two samples of sera from before the development of cancer were available for 118 cases with Kaposi's sarcoma and their matched controls. The mean time between the first and second blood samples was 766 days (standard deviation 350 days). At the time of first sample collection, the prevalence of latent anti-KSHV antibodies was 40% (47 out of 118) among those who developed Kaposi's sarcoma and 11% (13 out of 118) among their matched controls. In the second sample, the prevalence was the same for cases but higher for controls (40% (47 out of 118) *vs* 21% (25 out of 118)). Using those who were seronegative in both serum samples as a comparison group, the risk of Kaposi's sarcoma was significantly (*P*<0.05) higher among those people who were seropositive in the first sample, irrespective of their serostatus in the second sample (Table 4). There was no evidence that those who seroconverted to KSHV latent antibodies in the time between the first and second samples had a risk of Kaposi's sarcoma over and above that of those who remained seropositive throughout the study period. Similar results were obtained when additionally adjusted for CD4 count (data not shown).

Of the 118 case–control pairs with two samples of sera, for 116 there was sufficient volume of sera available to test for lytic anti-KSHV antibodies. At the time of first sample collection, the prevalence of lytic anti-KSHV antibodies was 23% (27 out of 116) among those who developed Kaposi's sarcoma and 15% (17 out of 116) among their matched controls. In the second sample, the prevalence was 22% (26 out of 116) among cases and 13% in controls. Using those who were seronegative in both serum samples as a comparison group, the risk of Kaposi's sarcoma was significantly (*P*<0.05) higher among all other groups (Table 4), although there was no evidence that those who seroconverted to KSHV lytic antibodies in the time between the first and second samples had a risk of Kaposi's sarcoma over and above that of those who remained seropositive throughout the study period. Similar results were obtained when additionally adjusted for CD4 count (data not shown).

In relation to antibodies against EBV-VCA, for a doubling of titre, the odds ratio for Kaposi's sarcoma was estimated to increase by a multiplicative factor of 1.1 (95% CI 0.9–1.2; *χ*^2^_1_=0.6, *P*=0.4; Table 5). After adjustment for CD4 count at the time of sample collection, this factor was 1.0 (95% CI 0.8–1.2; *χ*^2^_1_=0.04, *P*=0.8).

### Non-Hodgkin's lymphoma

The prevalence of antibodies against latent KSHV antigens was 15% (10 out of 67) in those with non-Hodgkin's lymphoma and 9% (6 out of 67) in their matched controls (OR=1.8, 95% CI 0.6–5.4; Table 6). The prevalence of antibodies against lytic KSHV antigens was 9% (six out of 66) in those with non-Hodgkin's lymphoma and 6% (four out of 66) in their matched controls (OR=1.5, 95% CI 0.4–5.3; Table 6).

Among all people with non-Hodgkin's lymphoma (67 case–control pairs; for those with two serum samples, the first was used), for a doubling of anti-EBV antibody titre, the odds ratio for non-Hodgkin's lymphoma was estimated to increase by a multiplicative factor of 1.3 (95% CI 0.9–1.7; *χ*^2^_1_=2.5, *P*=0.1; Table 5). After adjustment for CD4 count at the time of sample collection, the equivalent factor was 1.5 (95% CI 0.9–2.3; *χ*^2^_1_=3.30, *P*=0.1). Two samples of sera were available for 48 cases of non-Hodgkin's lymphoma and their matched controls. The mean time between first and second blood samples was 653 days (standard deviation 382 days). For 13 cases and six controls, the titre of antibodies against EBV stayed the same in the first and second samples; for 15 cases and 14 controls the titre declined between samples and for 18 cases and 26 controls the titre increased.

## DISCUSSION

Among HIV infected people, high levels of antibodies against KSHV are strongly predictive of the subsequent risk of Kaposi's sarcoma, but not of non-Hodgkin's lymphoma. We find no significant association between anti-EBV-VCA antibodies and non-Hodgkin's lymphoma or Kaposi's sarcoma in HIV seropositive adults.

Data from case–control studies (using the same assay against KSHV latent nuclear antigens encoded by orf73) suggest that high anti-KSHV antibody titres are associated with an increased risk of Kaposi's sarcoma both in HIV seropositive and seronegative people (Sitas *et al*, 1999; Davidovici *et al*, 2001; Newton *et al*, 2003a, 2003b; Ziegler *et al*, 2003). Prospective data are lacking, although a study of HIV infected people in Italy suggested that anti-KSHV antibody titres may be persistently high even before the diagnosis of Kaposi's sarcoma, based on the observation of 21 cases of the tumour (Rezza *et al*, 1999). Data presented here confirm, on substantially larger numbers of cases, that anti-KSHV antibodies are detectable before the diagnosis of the tumour and that the subsequent risk of Kaposi's sarcoma increases with increasing antibody titres (this is true for latent and lytic antibodies). Since all study participants were HIV seropositive at entry to the study, we could not investigate whether the risk of Kaposi's sarcoma was higher among those who seroconverted to KSHV after becoming infected with HIV as compared to before infection with HIV (Renwick *et al* 1998; Jacobson *et al*, 2000). However, we found no evidence that seroconversion to antibodies against KSHV during the course of this study was associated with a particularly increased risk of Kaposi's sarcoma as compared to people who had been seropositive for KSHV throughout.

Human immunodeficiency virus-associated non-Hodgkin's lymphomas are atypical and can be divided into three broad categories on the basis of their clinical, histological and epidemiological features. The most common subtype in almost all studies is a B cell immunoblastic lymphoma (Newton *et al*, 1999). Roughly half of such tumours have been found to contain evidence of EBV DNA in tumour tissue. Another subtype is Burkitt-type non-Hodgkin's lymphoma, which resemble the sporadic form of the disease; about 30% of these tumours have been found to contain EBV-DNA. The third major subtype is cerebral non-Hodgkin's lymphoma, almost 100% of which contains evidence of EBV DNA. Precise diagnostic information on the subtypes of non-Hodgkin's lymphoma was unavailable in this study.

In relation to non-Hodgkin's lymphoma, data from three prospective studies of people not known to be infected with HIV suggest that high antibody titres to the EBV VCA are predictive of an increased risk of the tumour. This is based on observation of 14 cases of Burkitt's lymphoma in Ugandan children and 115 cases of other non-Hodgkin's lymphomas in Europe and the USA (de-Thé *et al*, 1978; Mueller *et al*, 1991; Lehtinen *et al*, 1993). Results presented here are the only prospective data on anti-EBV antibody titres in relation to the development of lymphoma in people known to be infected with HIV.

As almost everyone studied was seropositive for antibodies against EBV, comparisons between cases and controls rely on a quantitative assessment of antibody titre, thereby reducing statistical power to detect true differences. Furthermore, recent work by Berrington de Gonzalez *et al* (submitted) demonstrated substantial variability in anti-EBV-VCA antibody titres for control samples, depending on laboratory conditions. The fact that non-Hodgkin's lymphoma comprises a heterogeneous group of diseases, only a proportion of which may be associated with EBV infection, coupled with variability in assay results, it is likely that a subtle association would be missed, particularly given the relatively small number of cases investigated here. Similarly, laboratory variation in results for antibodies against KSHV antigens may account for the discordant results found for a proportion of those individuals who had two samples tested.

In summary, among HIV infected people, high levels of antibodies against KSHV appear strongly associated with subsequent risk of Kaposi's sarcoma, but not with non-Hodgkin's lymphoma. Antibodies against EBV were not strongly associated with subsequent development of Kaposi's sarcoma or non-Hodgkin's lymphoma.

## Figures and Tables

**Table 1:** Distribution of Kaposi's sarcoma and non-Hodgkin's lymphoma cases and controls according to the matching variables

	**Kaposi's sarcoma**		**Non-Hodgkin's lymphoma**	
	**Cases (*N*=189)**	**Controls (*N*=189)**	**Cases (*N*=67)**	**Controls (*N*=67)**
*Sex*				
Males	189	189	61	61
Females	0	0	6	6
				
*Age (years)*				
<30	33	38	20	15
30–34	55	53	14	21
35+	101	98	33	31
				
*HIV transmission group*				
Homosexual	188	188	12	12
Other^a^	1	1	55	55
				
*Study*				
Concorde	67	67	20	20
Alpha	56	56	17	17
Delta	66	66	30	30
				
*Ethnicity*				
White	182	180	64	62
Other^b^	7	9	3	5
				
Mean time (days) between first blood sample and date of diagnosis of cancer/pseudo-diagnosis in controls	923 (standard deviation (s.d.) 725)	965 (s.d. 713)	957 (s.d. 786)	977 (s.d. 795)
				
*Time between 1st and 2nd sample (years)*				
<2	53	53	30	29
2+	65	65	18	19
NA^c^	71	71	19	19
				
*CD4 count (at 1st sample)*				
500–	33	45	4	14
200–	74	84	29	27
<200	56	34	17	9
NK^d^	26	26	17	17

a Heterosexual/i.v. drug user/haemophiliac/blood transfusion/other blood contact/other.

b Indian subcontinent/West Indian-Guyanan/African/other or mixed.

c NA: not applicable.

d NK: not known.

**Table 2:** Odds ratios (OR) for Kaposi's sarcoma according to a measure of latent and lytic anti-KSHV antibody titre

	**Unadjusted**		**Adjusted for CD4 count^a^**	
**Titre**	**Number of cases/controls *N*=189**	**OR (95% CI)**	**Number of cases/controls *N*=163**	**OR (95% CI)**
*Latent anti-KSHV antibodies*				
Negative	117/166	1.0	99/142	1.0
100–1600	27/11	3.9 (1.7–9.1)	25/10	3.5 (1.4–8.7)
3200–25 600	32/9	5.3 (2.3–12.5)	27/9	4.1 (1.7–10.2)
51 200+	13/3	5.7 (1.5–20.9)	12/2	8.7 (1.9–40.4)
		*χ* ^2^ *(1)=32, P<0.001*		*χ* ^2^ *(1)=26, P<0.001*
				

	**Unadjusted**		**Adjusted for CD4 count^c^**	
**Optical density^b^ (OD)**	**Number of cases/controls *N*=187**	**OR (95% CI)**	**Number of cases/controls *N*=161**	**OR (95% CI)**
*Lytic anti-KSHV antibodies*				
Negative	126/151	1.0	109/128	1.0
Low OD	18/10	2.2 (1.0–5.2)	14/10	1.2 (0.5–3.1)
Medium OD	26/13	2.5 (1.2–5.1)	24/12	2.8 (1.3–6.3)
High OD	17/13	1.4 (0.7–2.9)	14/11	1.4 (0.6–3.2)
		*χ*^2^*(1)=6.2, P*=*0.02*		*χ*^2^*(1)=4.3, P*=*0.04*

a Adjusted for CD4 count (<200, 200–500, 500+); the number of participants is fewer because some did not have a CD4 count available from around the time the blood was collected.

b Negative–optical density (OD) <0.84; low OD 0.84–1.06; medium OD 1.07–1.99 and high OD 2.0+.

c Adjusted for CD4 count (<200, 200–500, 500+).

**Table 3:** Odds ratios (OR) for Kaposi's sarcoma according to the latent (seropositive *vs* seronegative) and lytic (seropositive *vs* seronegative/indeterminate) anti-KSHV antibody status, stratified by the time between first sample collection and diagnosis of cancer

	**Unadjusted**			**Adjusted for CD4 count^a^**		
**Time between sample collection and diagnosis of Kaposi's sarcoma**	**No. of case–control pairs (total=189)**	**No. of seropositive cases/controls**	**OR (95% CI)**	**No. of case–control pairs (total=163)**	**No. of seropositive cases/controls**	**Adjusted OR (95% CI)**
*Latent anti-KSHV antibodies*						
<14 months	62	23/4	7.3 (2.2–24.5)	38	16/3	5.0 (1.4–18.5)
14–42 months	67	26/9	3.8 (1.6–9.4)	65	25/8	4.1 (1.5–11.4)
>42 months	60	23/10	4.3 (1.4–12.6)	60	23/10	4.1 (1.4–12.4)
			*χ*^2^*(1)=0.4, P*=*0.6*			*χ*^2^*(1)=0.04, P*=*0.8*
						

	**Unadjusted**			**Adjusted for CD4 count^a^**		
**Time between sample collection and diagnosis of Kaposi's sarcoma**	**No. of case–control pairs (total=187)**	**No. of seropositive cases/controls**	**OR (95% CI)**	**No. of case–control pairs (total=161)**	**No. of seropositive cases/controls**	**Adjusted OR (95% CI)**
*Lytic anti-KSHV antibodies*						
<14 months	60	16/6	2.7 (1.0–6.8)	36	11/4	3.9 (0.9–16.1)
14–42 months	67	17/11	1.8 (0.7–4.2)	65	17/10	2.6 (0.9–7.5)
>42 months	60	11/9	1.3 (0.5–3.5)	60	11/9	1.4 (0.5–3.9)
			*χ*^2^*(1)=1.1, P*=*0.3*			*χ*^2^*(1)=1.4, P*=*0.2*

a Adjusted for CD4 count (<200, 200–500, 500+); the number of participants is fewer because some did not have a CD4 count available from around the time the blood was collected.

**Table 4:** Odds ratios for Kaposi's sarcoma among those with two serum samples available for testing, according to whether the first and second sample was sero-positive or negative for latent and lytic anti-KSHV antibodies

	**Latent antibodies**		**Lytic antibodies**	
**1st sample/2nd sample**	**Number of cases/controls *N*=118**	**OR (95% CI)**	**Number of cases/controls *N*=116**	**OR (95% CI)**
neg/neg	56/89	1.0	71/92	1.0
neg/pos	15/16	1.5 (0.6–3.5)	16/7	3.8 (1.4–10.0)
pos/neg	15/4	9.6 (2.1–43.5)	17/8	2.7 (1.2–6.0)
Pos/pos	32/9	5.5 (2.6–11.6)	12/9	2.2 (0.9–5.1)
		*χ* ^2^ *(3)=27.9, P<0.001*		*χ*^2^*(3)=11.0, P*=*0.01*

**Table 5:** Multiplicative factor by which the odds ratios for Kaposi's sarcoma and non-Hodgkin's lymphoma increased in relation to a doubling of the anti-EBV antibody titre

	**Factor (95% CI)**	**Adjusted factor (95% CI)**
Kaposi's sarcoma	1.1 (0.9–1.2)	1.0 (0.8–1.2)
Non-Hodgkin's lymphoma	1.3 (0.9–1.7)	1.5 (0.9–2.3)

**Table 6:** Odds ratios for non-Hodgkin's lymphoma in relation to latent and lytic antibodies against KSHV

	**Unadjusted**		**Adjusted for CD4^a^**	
**Titre**	**Number of cases/controls *N*=67**	**OR (95% CI)**	**Number of cases/controls *N*=50**	**OR (95% CI)**
*Latent anti-KSHV antibodies*				
Negative	57/61	1.0–	43/44	1.0–
Positive	10/6	1.8 (0.6–5.4)	7/6	1.2 (0.3–5.3)
		*χ*^2^*(1)=1.2, P*=*0.3*		*χ*^2^*(1)=0.04, P*=*0.8*
				

	**Unadjusted**		**Adjusted for CD4^a^**	
**Titre**	**Number of cases/controls *N*=66**	**OR (95% CI)**	**Number of cases/controls *N*=49**	**OR (95% CI)**
*Lytic anti-KSHV antibodies*				
Negative	60/62	1.0–	44/45	1.0–
Positive	6/4	1.5 (0.4–5.3)	5/4	0.5 (0.1–2.7)
		*χ*^2^*(1)=0.4, P*=*0.5*		*χ*^2^*(1)= 0.6, P*=*0.4*

a Adjusted for CD4 count (<200, 200–500, 500+); the number of participants is fewer because some did not have a CD4 count available from around the time the blood was collected.
